# I-RREACH: an engagement and assessment tool for improving implementation readiness of researchers, organizations and communities in complex interventions

**DOI:** 10.1186/s13012-015-0257-6

**Published:** 2015-05-04

**Authors:** Marion Maar, Karen Yeates, Marcia Barron, Diane Hua, Peter Liu, Margaret Moy Lum-Kwong, Nancy Perkins, Jessica Sleeth, Joshua Tobe, Mary Jo Wabano, Pamela Williamson, Sheldon W Tobe

**Affiliations:** Faculty of Medicine, Northern Ontario School of Medicine, Laurentian University, Sudbury, ON Canada; Department of Medicine, Queens University, Kingston, ON Canada; Riverstone Research Consulting, Guelph, ON Canada; Department of Nephrology, Sunnybrook Health Sciences Centre, Sunnybrook Research Institute, University of Toronto, Toronto, ON Canada; University of Ottawa Heart Institute, Ottawa, ON Canada; Department of Research, Advocacy and Health Promotion, Heart and Stroke Foundation of Ontario, Toronto, ON Canada; Department of Medicine, Western University, London, ON Canada; Wikwemikong Health Centre, Wikwemikong, ON Canada; Noojmowin Teg Health Centre, Little Current, ON Canada

**Keywords:** Complex intervention, Community-based participatory research, Methodology, RCT, Implementation tool, International health, Aboriginal health, LMIC, Canada, Tanzania

## Abstract

**Background:**

Non-communicable chronic diseases are the leading causes of mortality globally, and nearly 80% of these deaths occur in low- and middle-income countries (LMICs). In high-income countries (HICs), inequitable distribution of resources affects poorer and otherwise disadvantaged groups including Aboriginal peoples. Cardiovascular mortality in high-income countries has recently begun to fall; however, these improvements are not realized among citizens in LMICs or those subgroups in high-income countries who are disadvantaged in the social determinants of health including Aboriginal people. It is critical to develop multi-faceted, affordable and realistic health interventions in collaboration with groups who experience health inequalities. Based on community-based participatory research (CBPR), we aimed to develop implementation tools to guide complex interventions to ensure that health gains can be realized in low-resource environments.

**Methods:**

We developed the I-RREACH (Intervention and Research Readiness Engagement and Assessment of Community Health Care) tool to guide implementation of interventions in low-resource environments. We employed CBPR and a consensus methodology to (1) develop the theoretical basis of the tool and (2) to identify key implementation factor domains; then, we (3) collected participant evaluation data to validate the tool during implementation.

**Results:**

The I-RREACH tool was successfully developed using a community-based consensus method and is rooted in participatory principles, equalizing the importance of the knowledge and perspectives of researchers and community stakeholders while encouraging respectful dialogue. The I-RREACH tool consists of three phases: fact finding, stakeholder dialogue and community member/patient dialogue. The evaluation for our first implementation of I-RREACH by participants was overwhelmingly positive, with 95% or more of participants indicating comfort with and support for the process and the dialogue it creates.

**Conclusions:**

The I-RREACH tool was designed to (1) pinpoint key domains required for dialogue between the community and the research team to facilitate implementation of complex health interventions and research projects and (2) to identify existing strengths and areas requiring further development for effective implementation. I-RREACH has been found to be easily adaptable to diverse geographical and cultural settings and can be further adapted to other complex interventions. Further research should include the potential use of the I-RREACH tool in the development of blue prints for scale-up of successful interventions, particularly in low-resource environments.

**Electronic supplementary material:**

The online version of this article (doi:10.1186/s13012-015-0257-6) contains supplementary material, which is available to authorized users.

## Background

Non-communicable chronic diseases such as cardiovascular disease (CVD), cancers, diabetes and chronic lung diseases are the leading causes of mortality globally, accounting for approximately two thirds of annual deaths. Nearly 80% of these deaths occur in low- and middle-income countries (LMIC) [1]. In high-income countries (HIC), inequitable distributions of resources affect poorer and otherwise disadvantaged groups including Aboriginal peoples [2,3]. Furthermore, LMICs are faced with large competing burdens of communicable diseases and fragmented, poorly-resourced health care systems that result in significant challenges in managing the complexities of multiple risk factors that attribute to chronic non-communicable diseases.

Among chronic diseases, cardiovascular disease is the number one cause of mortality worldwide. However, cardiovascular mortality in high-income countries has recently begun to fall which can be attributed to both advances in treatment as well as control of risk factors using evidence-based management models [4,5]. Making these advances accessible to all should be a priority globally. While HICs are effectively reducing cardiovascular mortality [6], the gains are not realized among citizens in LMICs nor in those subgroups in HICs who are disadvantaged in the social determinants of health (SDOH) such as Aboriginal people [1]. As chronic diseases such as hypertension and diabetes continue to rise in LMICs and Aboriginal populations in Canada, it is critical to develop multi-faceted, affordable and realistic health interventions informed by an understanding of local SDOH in order to ameliorate them and improve health equity [1,7].

### Barriers to CVD and hypertension treatment in LMICs and Aboriginal communities

The availability of clinical practice guidelines for cardiovascular disease is necessary but not sufficient alone to bring about significant improvements in cardiovascular health. Risk factor reduction is complex and requires lifestyle modifications, behaviour changes and pharmacologic therapy. Health care policies may actually contribute additional barriers to evidence-based care and changes to health systems, and these policies may be required to improve the prevention and management of chronic diseases [8].

Facing the mounting burden of CVD and related complications, LMICs and Aboriginal populations often lack the financial means and/or geographically accessible services for diagnosis, treatment and management options, leading to increased rates of morbidity and mortality [1]. In Canada for example, studies show that death rates from circulatory system diseases among Aboriginal people are significantly elevated compared with non-Aboriginal people [9]. Hypertension, one of the major risk factors for CVD, is highly prevalent and has no noticeable symptoms; therefore, it is too often undiagnosed and goes untreated in low-resource and low access health care environments where the focus is on other more urgent or competing medical problems. A recent systematic review in Canadian Aboriginal communities found an average prevalence for hypertension of 23.5% [10]. High rates of undiagnosed hypertension [11] were also linked to elevated rates of chronic kidney disease [12]. Barriers to care include lower access to renal dialysis and transplantation [13].

Although geographically and culturally quite dissimilar to Canadian Aboriginal populations, resource-poor African countries such as Tanzania struggle with similar predicaments. While there are no national data sets on the rates of hypertension, current evidence points to higher hypertension prevalence with dramatically lower treatment and control rates [14] associated with higher stroke rates [15]. Compared to rural and remote Canadian communities, the lower access to hypertension management in Tanzania is due, in large part, to financial resource constraints limiting numbers of adequately trained health care providers, diagnosis and treatment as well as a lack of infrastructure connecting patients, their medical records and their health care providers [16].

### DREAM-GLOBAL: a community-based hypertension intervention for LMIC and Aboriginal communities

**Figure 1:**
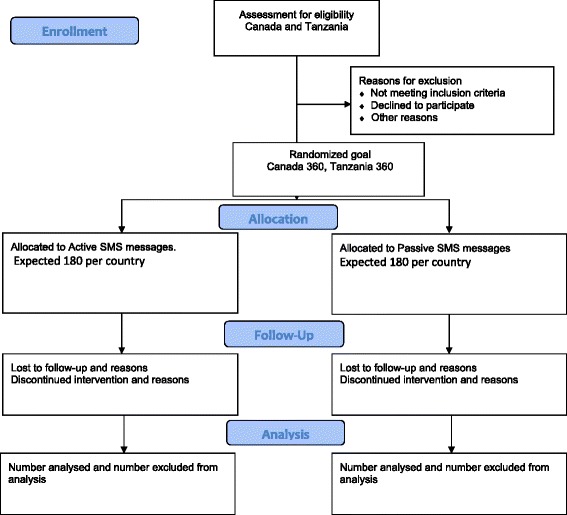
DREAM-GLOBAL consort diagram.

DREAM-GLOBAL (Diagnosing hypeRtension-Engaging Action and Management in Getting LOwer Bp in Aboriginal and LMIC) is a research project designed to increase our knowledge of how to develop and implement affordable, evidence-based, guidelines-driven hypertension management interventions at the patient, provider and community level in LMICs (Tanzania) and Aboriginal communities in Canada by leveraging innovative technologies, health services and research methodology. The project is informed by the Canadian Hypertension Education Program (CHEP) clinical practice guidelines drawing on evidence for high blood pressure prevention through dietary sodium restriction, blood pressure measurement, education interventions for health care providers and patients, inter-professional care, health systems and community-based interventions including automated reminder systems [17,18]. It combines a multilayered approach including many elements of the chronic care management model, as well as task shifting from health care providers (HCP) to community health resource and community health workers. The project also facilitates self-management support, decision support, delivery system design, clinical information systems, while building on existing capacity in health care organizations and community resources [19]. DREAM–GLOBAL uses a randomized controlled trial (RCT) [20] design to test the effectiveness of a mobile health strategy using cell phone-based *short message service* (SMS) technologies to facilitate blood pressure measurements and feedback between patient and provider through the local existing mobile phone network. The flow diagram in Figure 1 displays the planned progression of participants through the trial. The study is a complex intervention leading to changes in the behaviour of patients, providers and local systems. It is implemented using a collaborative community-based participatory approach.

### Informing complex interventions through community-based participatory research

The components of the DREAM-GLOBAL intervention are evidence-based, but we recognize that interventions can easily fall short of achieving the intended outcomes due to ineffective implementation and delivery in their ‘real life’ application within the health care system. In particular, low-resource environments are strongly affected by implementation issues. This phenomenon, known as the implementation gap, is linked to the lack of understanding of the multifarious factors that may impact on the effectiveness of an intervention when implemented at the community or population level [21]. Confounding factors include the uniqueness of local power structures, health systems, community organizations and human actors making standardization unrealistic.

There is an increasing acknowledgement that to measure the degree of effectiveness of an intervention in real clinical practice, an RCT must be pragmatic, maximizing external validity (generalizability) while trying to preserve internal validity (reliability and accuracy) [22]. A traditional RCT design does not offer an analysis of the multiple factors that impact on outcomes nor how or why the observed impact occurred [23]. Leykum and colleagues argue that this leads to a profound dilemma: “How do we design interventional trials that are generalizable, but also have enough flexibility to be meaningful and more likely to be successful locally?” [24] Research leading to a better understanding of key implementation factors and the mechanism by which these influence outcomes are needed to guide implementation strategies for intervention trials as well as proven interventions in diverse environments.

Given the rising rates of CVD in disadvantaged populations, it is essential to study the implementation factors of CVD interventions in low-resource environments where they are urgently needed rather than extrapolating from high-income settings [21] The DREAM-GLOBAL intervention trial is planned for diverse communities in Tanzania and Aboriginal communities in Canada, and we therefore explored these key implementation issues through participatory research prior to rolling out the study. Our objective was to use the information gathered to develop an implementation tool to guide the process of identifying key implementation issues related to people, community, health systems, culture and relationships that are likely to differ between settings. As each of these factors introduces complexity, our goal was to design the implementation tool to be easily adaptable to various health care environments and health issues in a variety of communities globally.

Using community-based participatory research (CBPR), we aimed to develop a tool to guide dialogue between researchers and communities on key aspects of researcher, community, system functionality and readiness. We report on (1) the theoretical underpinnings of this tool, (2) the key domains of complexity and issues and how these are uncovered by this tool, and (3) the evaluation feedback from stakeholders during the testing of the tool.

### Methodology

Our methodology for the development of the tool consisted of a three-pronged CBPR approach: We employed a consensus method to (1) develop the theoretical basis of the tool and (2) to identify key implementation factor domains; then, (3) we evaluated the tool.

### A community-based participatory research approach

CBPR is not a research methodology but rather a value-based approach to inquiry. It has been defined as “a collaborative research approach that is designed to ensure and establish structures for participation by communities affected by the issue being studied, representatives of organizations, and researchers in all aspects of the research process to improve health and well-being through taking action, including social change” [25]. Israel and colleagues articulate several key principles that define CBPR. These principles include notions such as recognizing the community as a unit of identity, building on community strengths, collaboration in all phases of the research, integration of research and action based on co-learning and knowledge sharing to address inequalities [26].

Translational and intervention research in low-resource environments frequently has a distinct cross-cultural aspect, with much of the scientific evidence provided by cultural outsiders. Rivkin and co-workers argue that this can pose challenges for intervention science as health concepts used in scientific research may not translate or have different meanings in culturally diverse partner communities [27]. Trickett and others have further elaborated that “culture pervades all aspects of community interventions” and community collaboration are fundamental to the success of complex interventions [28]. Warry noted that collaborative research in these settings requires researchers to relinquish control over the research process in order to strengthen their explanations [29].

In this study, the CBPR approach formally began when community stakeholders interested in exploring the implementation of DREAM-GLOBAL were invited to share their perspective on the development of an implementation tool based on their lived experience within partner communities using a consensus method.

### Indigenous consensus method approach to the development of the theoretical basis and domains of the implementation tool

A consensus method informed by Indigenous values [30] and CBPR principles was used to initiate a dialogue involving academic researchers and community stakeholders addressing potential implementation issues for DREAM-GLOBAL. Community stakeholders consisted of people with lived experience and close connections in the targeted partner communities. They included health care workers and administrators, community-based researchers, health champions and those who were or had family affected by hypertension.

The Indigenous consensus method that we employed differs from traditional academic consensus methods such as the Delphi method [31] as it does not privilege the perspective of academic experts. Instead, the process invites cultural values and knowledge gained from the lived experience of participants, by eliciting the perspectives of those who are eventually affected by the intervention such as community members and local communities of practice. The consensus process is completed once the nexus between community, academic and practice-based knowledge manifests, which in this case was achieved after three consensus cycles (see Table 1).

**Table 1:** **Participatory consensus cycles employed in the development of I-RREACH**

**Consensus cycle**	**Canada**	**Tanzania**	**Theoretical frameworks tested**	**Theoretical perspectives identified as relevant**
Cycle 1: discussion of draft concepts	July 2012, DREAM-GLOBAL annual meeting; facilitated stakeholder discussion	March 2013, group of 16 community-based researchers	Researchers’ practice-based knowledge, Community Readiness Assessment	CBPR, Indigenous approaches to research, empowerment approaches
Cycle 2:	Discussions with key stakeholders during community-based engagement visits	November 2013, group of 11 community-based researchers	CBPR, Indigenous approaches to research, empowerment approaches	CBPR, Indigenous approaches to research, empowerment approaches, practice-based knowledge
Cycle 3	Invitation to Aboriginal community health staff for written feedback on draft tool	November 2013, key informant community researchers	Community-based collaborators’ practice-based knowledge.	None identified, consensus on applicable theoretic frameworks was achieved at the end of cycle 3

#### First consensus cycle

Based on the academic research team’s past experience (i.e. their practice-based knowledge) in implementing interventions in a wide range of community settings, an initial set of key factors that were reasoned to impact implementation was created in order to provide discussion materials for the first meeting with community collaborators. These factors included the following: (1) community engagement; (2) local health care delivery system; (3) patient self- management; (4) health policy; (5) available infrastructure, including information and communication technology; and (6) historical, social and cultural issues. These domains were then refined and theoretically contextualized within the literature on community readiness assessment [32-34] to develop the first list of draft domains and proposed approach to assessing readiness.

The first consensus cycle to elicit community feedback on the draft domains and research approach was then initiated. In Canada, it took the form of a workshop meeting involving a group of invited stakeholders from Aboriginal communities interested in exploring the implementation of the project in their community in dialogue with Aboriginal health and clinical researchers. In Tanzania, the workshop included community members, local community researchers and academic researchers. The dialogue was a rich exchange of lived and technical experience in both the Canadian and Tanzanian environments. Participants provided feedback on implementation factors and the conceptual framework of implementation readiness assessment. Aboriginal and Tanzanian stakeholders provided significant direction for revisions to the theoretical orientation of the framework and commented on the relative importance of the draft domains.

#### Second consensus cycle

The second loop was designed to validate key implementation issues and a revised theoretical framework (based on feedback from the first cycle) through member checking with additional stakeholders during various community meetings at sites in Canada and Tanzania. The researchers gathered detailed feedback on the revised tool, its implementation, as well as culturally competent phrasing of questions and probes.

#### Third consensus cycle

The third feedback loop was designed to gain a final member check on the key issues, domains, questions, probes and implementation process of the tool. The focus in this round was to include members of the community health care staff in Canada and Tanzania. This cycle provided only minor feedback on phrasing. However, the name of the implementation tool was developed during this final round.

### Participant feedback on the I-RREACH tool

The finalized I-RREACH (Intervention and Research Readiness Engagement and Assessment of Community Health Care) tool is an implementation tool that includes a community fact sheet, an interview, and focus group guide designed to elicit information on key factors that may impact the implementation of the intervention. It was implemented during visits to participating communities in Canada and Tanzania. At the end of each interview and focus group, participants were asked to complete a short anonymous evaluation form rating their immediate experience with the I-RREACH process. The evaluation tool was designed to gather information on a five-point scale on how participants perceived the following: (1) the clarity of questions, (2) rapport and understanding between researcher and themselves, (3) improvements in understanding of the intervention and implementation issues, (4) cultural safety, (5) I-RREACH process as a facilitation tool for knowledge exchange for implementation of the intervention and (6) any comments or additional suggestions for improvements. The evaluation form is provided in Table 2. It is scaled to indicate high levels of satisfaction when participants either “agree” or “strongly agree” with the provided statements. It can be easily adapted to I-RREACH evaluations by researchers who wish to use this tool in the future. To summarize the data, we tallied up scores for each of the five scored domains and thematically summarized the short responses and suggestions for improvements. While any dissatisfaction is reason for concern that researchers may need to explore, we deemed the I-RREACH tool to be acceptable from the participants’ perspective if at least 85% of scores were in the combined “agree” and “strongly agree” categories and no major concerns were raised in the open-ended question component of the questionnaire.

**Table 2:** **Participant evaluation form for the I-RREACH tool**

**DREAM-GLOBAL: Intervention and Research Readiness Engagement and Assessment of Community Health Care (I-RREACH) feedback form for participants**					
**Your feedback would be appreciated regarding the focus group/interview sessions for the I-RREACH project of the DREAM-GLOBAL Study. This feedback is very important for future planning and use of this tool and process in other communities. We would appreciate you taking the time to complete this evaluation form and returning it at the end of the meeting.**					
**Content evaluation (check one only)**					
	**Strongly disagree**	**Disagree**	**Neutral**	**Agree**	**Strongly agree**
1. The questions asked were clear and made sense to me.					
2. I think the researcher understood my perspective (two-way exchange of information).					
3. After attending this focus group/interview, I have a better understanding of the DREAM-GLOBAL project goals and how it can be implemented.					
**Cultural safety evaluation (check one only)**					
4. I felt comfortable with what we discussed during the focus group/interview.					
Please Explain:					
5. The focus group/interview was a good way to exchange information and ideas related to the project.					
Please Explain:					
6. What did you like best about the session?					
7. Is there anything you think we should change?					

### Ethics review

In line with the community-based participatory nature of the development of the I-RREACH tool, the study protocol was submitted for academic ethics review and community-based REB review (in Canada). Members of the research team also sought formal approval by decision makers in each of the participating communities after local, in-person presentations. Academic and governmental ethics approvals include the following: The National Institute for Medical Research in Dar es Salaam, Tanzania (approved March 19, 2014); Queen’s University Health Sciences and Affiliated Teaching Hospitals Research Ethics Board, Kingston, Ontario (DMED-1603-13 approved June 21, 2013); and Sunnybrook Health Sciences Centre Research Ethics Board, Toronto, Ontario, (#182-2013, approved May 31, 2013). Community-based ethics review in Aboriginal communities included The Cree Board of Health and Social Services of James Bay, Ontario (approved September 11, 2013) and Manitoulin Anishinaabek Research Review Committee (MARRC) Ontario (approved October 7, 2013). The study was also formally approved by decision making bodies in all participating communities through Village Councils approvals in Tanzania and Band Councils Resolutions in First Nations in Canada.

## Results

In this section, we present (1) the theoretical basis of the I-RREACH tool and its implementation; (2) the three components of I-RREACH, its information domains as well as the finalized version of the I-RREACH tool tailored to DREAM-GLOBAL; and (3) participant evaluations of I-RREACH during implementation.

### Resolving theoretical dissonance through consensus cycles

#### Results of the first consensus cycle

The Community Readiness Assessment (CRA) approach was first explored by the academic researchers as a lens to guide the development of the implementation tool. CRAs presuppose that the locus of change is solidly anchored within the community, with researchers assessing how much the community has “to be moved though stages of change” based on the transtheoretical model of change [33]. Further, the CRA emphasizes a predominantly quantitative approach that leads to ranking of communities (based on scores from ratings scales) with respect to their research readiness measured by parameters designated by the researchers. During the first consensus cycle however, stakeholders strongly advocated for a need to incorporate a community empowering CBPR approach as well as respect and understanding for Indigenous and community research values into the design of the implementation tool. CRA was seen as dissonant with the aspirations of community stakeholders, because it ignores potential capacity gaps and changes required in the academic research team, health systems and policies that may be essential to make an intervention work in a given community. Community stakeholders therefore strongly rejected the CRA approach for DREAM-GLOBAL, as they perceived it and its use of rankings as unrealistic and paternalistic. While it is important as it has been argued in the CRA approach that communities pull together in the development of interventions [35], our CBPR showed that this is just one of many key issues that relates to effective implementation. Our new understandings of the multiple factors that may impact on implementation in communities in low-resource environments were incorporated into our implementation tool.

Another theme which emerged during the first consensus cycle was that Aboriginal and Tanzanian research participants preferred to move away from disempowering outsider-based assessments of communities centered on what outside experts deemed as important towards a dialogue and knowledge exchange to co-create a shared understanding of implementation issues in Aboriginal and Tanzanian communities.

#### Results of the second and third consensus cycles

Based on community feedback in the first consensus cycle, a new goal was articulated to develop a qualitative implementation tool with the following characteristics: (1) to employ the principles of CBPR and affirming practice-based knowledge and Indigenous lived experience; (2) to enhance bidirectional learning between researcher and community stakeholders as well as their collective understandings of the individual, social and organizational determinants that may affect the intervention; (3) to identify existing strengths and empowering approaches that may support implementation; as well as (4) to recognize areas where additional assistance may be required by researchers, communities and systems to enable effective implementation.

The consensus dialogue provided the impetus for a substantial theoretical shift of the tool from its first iteration as a researcher-driven, unidirectional assessment of community capacity to its final version as a collaborative, knowledge exchange and dialogue tool. As a result, alternative theoretical models had to be explored as the basis for the implementation tool and newly adjusted theoretical underpinnings incorporated into the tool were shared during the second feedback loop (see Table 1). The relevance of the CBPR and affirmation of collaboration in all aspects of implementation, as well as the need for capacity building with communities and academic researchers was confirmed. Minor adjustments were made and specific questions in each domain were developed for different audiences in the community. An interview guide for health care providers was developed with a complementary focus group tool for community members as well as a fact-finding sheet designed to gather community specific information and contacts. The name of the tool was then changed from a ‘Community Readiness Assessment Tool’ to the Intervention and Research Readiness, Engagement and Assessment of Community Health Care Tool (I-RREACH) to reflect the commitment to a participatory approach and bidirectional knowledge exchange.

### Components and information domains of the finalized I-RREACH tool

The information gathering and dialogue tool we developed consists of three stages: (1) a community profile section with a fact table which can be populated through various fact-finding activities, (2) an interview guide to help facilitate the discussion and understanding of strategic topics with key stakeholders in the community and (3) a focus group guide to lead a dialogue on community-centered issues.

### I-RREACH phase 1: community profile (fact table)

This section of the I-RREACH tool is designed to be employed at the start of the engagement phase. This section gathers relevant community profile information to determine if a specific community has the basic characteristics that would enable implementation of the intervention. Information gathering is focused on the community, relevant governance, health care staff, structure and programs, infrastructure and technology as well as resources. This data may be found in publically available records and during initial contacts with the community. When this part of the I-RREACH tool is used, it may not be clear yet if the implementation will occur, either because the community has not yet fully affirmed interest or because certain characteristics identified in the community profile may exclude the community from participation. For example, in the DREAM-GLOBAL study, cell phones are used to send educational SMS text messages to patients and lack of cell phone coverage would therefore disqualify a community from participation. Other studies may require specific health care capacities or community demographics, all of which would be identified during this early data collection phase.

### I-RREACH phase 2: key informant perspective (interview guide)

During this phase of I-RREACH, a dialogue with key stakeholders is established. We learned that key stakeholders should be identified early on in collaboration with the local contacts. Key stakeholders generally are individuals who could have a negative or positive effect on the implementation of the intervention. Many of these key stakeholders may be affected by the intervention through training, changes in practice or additional responsibilities. Others may be decision makers, health champions or people personally affected by the health issue or they may wield influence in the community. The discussion sessions with key stakeholders in this phase builds on the information gathered in the community profile but elicits the more practical and community-specific knowledge that is needed for effective implementation from an operational perspective. The interview phase also allows stakeholders to learn about the intervention and reflect on their expectations and level comfort with the project. The interview domains cover characteristics of local leadership, descriptions of services, access and awareness of relevant community programs, local understanding of the health issues, resources and planning, the perceived fit between the intervention and community goals and finally the requirements and preparedness of researchers and community to conduct collaborative research and implementation of the intervention. Interviewers should continually probe for information that would lead them to a higher level of understanding of the role of politics, policy and resource allocation on the health issue in question; the cultural context and community life; local interaction of medical traditions; oppressive hegemonic experiences in the community with health services or research; and community expectations related to the research and the academic team.

### I-RREACH phase 3: community members perspective (focus group guide)

The focus group discussions with community members cover similar issues as the topics discussed in the previous phase; however, the guide is designed to elicit the lived experience of those who are expected to be the main recipients of the intervention. The discussion framework explores the qualities of community life and history and how the health issue is culturally situated, such as the intertwining of health and social issues as well as expectations of community members. Providing a safe environment to exchange information about the intervention and inviting reflection on community members’ expectations and level comfort with the initiative and research team’s readiness to work with the community is an important requirement in this phase.

The domains of information covered in each of the I-RREACH phases are summarized in Table 3. Using the identified key domains, a comprehensive tool was developed to gather information and initiate dialogue. The complete tool adapted to the hypertension management intervention in Aboriginal communities in Canada and rural villages in Tanzania as part of the DREAM-GLOBAL RCT is provided in Additional file 1. The tool can be easily adapted to support implementation of interventions for other health issues in various environments.

**Table 3:** **I-RREACH components with listing of respective information domains**

**Information Domain**	**(Phase 1)**	**(Phase 2)**	**(Phase 3)**
	**Community profile tool (document and internet based)**	**Key informant perspective (interview guide)**	**Community members perspective (focus group guide)**
Basic community descriptions	Demographic information	n/a	n/a
Leadership	Basic contact information	Formal and informal leadership; economic and political structures	n/a
Community programs	Contact information, addresses, organizational information	Description of activities, quality, cultural relevance, integration of services, community awareness and access; interacting medical traditions	Lived experience of the health issues in the community, cultural context, program quality, perspectives on self-management, cultural perspectives; interacting medical traditions
Local understanding of the health issue	n/a	Perceived importance and quality of local health data on the issue	Pathways to access to health information
Resources and planning	Basic descriptions of funders and initiatives	Implications of funding streams and planned initiatives	n/a
Perceived fit of the intervention with community objectives	n/a	Past experiences with similar interventions, potential challenges and facilitators	Context of culture and community, challenges and facilitators; medical traditions
Infrastructure and technology	Basic descriptions	Community comfort, use of technology, barriers	Community comfort, level of use in the community and with different groups
Readiness for community-based research	n/a	Quality of community experience with research; community expectations; competency and learning requirements for researchers	Quality of community experience with research; community expectations; competency and learning requirements for researchers; experience of past oppression

Gathering information on key implementation domains from different sources as suggested in the I-RREACH tool allows for rigorous data collection, including identification, triangulation and member checking of implementation issues. The collected information helps implementation teams to build on existing community strengths and anticipate and ameliorate barriers to program implementation. The rich qualitative data from key informants as well as the lived experience of community members allows researchers to gain a deeper understanding of practical implementation aspects and community expectations. This, in turn, allows researchers to reduce their knowledge gaps and respond to local needs more effectively. Further, by engaging in meaningful dialogue, it is hoped that unexpected miscommunications or errors made along the way will be similarly communicated within the team of academic and community members for quick resolution.

### Evaluation results of the I-RREACH tool

The I-RREACH tool was implemented during community visits in five Aboriginal Communities in Canada and in two communities in Tanzania. A total of 135 informants participated in twelve focus groups and seven interviews in Canada and Tanzania. Modest incentives were provided for participants and included a catered meal and small monetary compensation in recognition of participant’s time and incurred expenses. Translators were present as required. An interactive education session on hypertension tailored to the audience was offered prior to the focus groups.

After their participation in I-RREACH focus group or interview sessions, 83 participants completed an evaluation of the I-RREACH session. When asked to assess the content of the I-RREACH dialogue, most of these participants (98.8%) agreed or strongly agreed that the interview and focus group questions were clear, helped the researcher to understand their perspective (90.4%) and enhanced their understanding of the project (95.2%). From the perspective of cultural safety, 95.2% agreed or strongly agreed that they felt comfortable with the I-RREACH sessions and 97.6 % agreed or strongly agreed that the I-RREACH sessions were a good way to exchange ideas between the research team and the community stakeholders. Table 4 provides the evaluation tool and a summary of the relative scoring completed by participants in this study.

**Table 4:** **Participant evaluation tool for I-RREACH with tallies of scores and percentages of participants choosing each score**

**Participant evaluation of I-RREACH**					
**Content evaluation**					
	**Strongly disagree**	**Disagree**	**Neutral**	**Agree**	**Strongly agree**
1. The questions asked were clear and made sense to me.	0	0	1	32	50
	0.0%	0.0%	1.2%	38.6%	60.2%
2. I think the researcher understood my perspective.	1	2	4	38	37
	1.2%	2.4%	4.8%	45.8%	44.6%
3. After attending this focus group/interview, do you have a better understanding of the DREAM-GLOBAL project and how it can be implemented?	0	1	3	39	40
	0.0%	1.2%	3.6%	47.0%	48.2%
**Cultural safety evaluation**					
4. I felt comfortable with what we discussed during the focus group/interview.	0	2	2	29	50
	0.0%	2.4%	2.4%	34.9%	60.2%
5. The focus group/interview was a good way to exchange information and ideas related to the project.	1	0	1	43	38
	1.2%	0.0%	1.2%	51.8%	45.8%

Based on an analysis of the written answers to open-ended questions in the evaluation, we also learned that the vast majority of participants were comfortable with what was discussed during the focus group sessions and also why people felt this way. The most common explanations provided included that they enjoyed learning about high blood pressure and its management and they appreciated the positive and respectful demeanor of the presenting research team members. This underscores the value of including engaging education activities in community visits. Many participants stated that the informal atmosphere made them feel comfortable and at ease. Many appreciated the opportunity to share their views and experiences of high blood pressure with others and to listen to others’ perspectives in the friendly and empowering atmosphere.

Most participants indicated that no changes for I-RREACH were needed; however, based on the content analysis of the open-ended questions, some stressed the importance of ongoing close contact between the research team and participants, requirements for training opportunities for staff and support for potential participants who struggle with healthy lifestyles due to difficult economic circumstances. Their recommendations are in line with the CBPR approach.

## Discussion

### A focus on exchange of practical and technical knowledge

According to Buchannan [36], the most widely shared definitions of theory in health sciences are still based on the positivist ideas that a theory should be testable and generalizable. While this positivist model is adequate to explain and predict biological aspects of health problems, the complexity of human practices and actions and variability in the characteristics of health care settings makes a natural sciences model much less appropriate for health care research.

Researching not only *if* but also *how* an intervention has an impact on change on the other hand provides more useful knowledge and allows weak links in the causal chain to be strengthened during the implementation. The UK’s Medical Research Council’s guidelines [37] for developing and evaluating randomized controlled trials for complex interventions emphasize the importance of understanding how and why an intervention works.

“The rationale for a complex intervention, i.e. what changes are expected, and how change is to be achieved, may not be clear at the outset. If so, a vitally important early task is to develop a theoretical understanding of the likely process of change, by drawing on existing evidence and theory, supplemented if necessary by new primary research, for example interviews with stakeholders ”[23].

Similarly, Green argues that theory in health research should not be considered as “offering universal explanations or predictions, but rather as enhancing understanding of complex situations. Such understanding will inevitably need to be sensitive to specific contextual factors, and would necessarily draw on the experience of practitioners and communities” [38]. The notion of practice-based knowledge (or praxis) focuses on what we have learned through experience on how to move from theoretical knowledge to practice at the point of intervention [39]. Aristotle is credited with advancing the idea of praxis as knowledge which is based on experiences with social and historical relationships which correspond to “what we might call wisdom today” [36].

The notions of praxis and importance of community-held wisdom for implementation of a complex intervention became important theoretical underpinnings contributing to the development of the I-RREACH tool, as the team drew on researcher and community stakeholder experience to recognize the domains that may impact on the effectiveness of interventions in Aboriginal and Tanzanian communities. Furthermore, community stakeholders firmly emphasized the need for bidirectional learning, dialogue and ongoing reflection related to project implementation, which will provide ongoing information to the research team on *how* the intervention is working or *why* it is not. Similarly, Leykum and colleagues have advocated for the integration of local sensibilities and ongoing reflection into pragmatic research design [24]. Based on our experience thus far with DREAM-GLOBAL, practical reasoning can provide a critical lens to focus our inquiry to recognize and respond to insights based on the unique community and organizational context, circumstances and challenges as well as historical and cultural dimension that may affect the intervention.

In order to establish good working relationships, it is essential that university and community-based collaborators have an in-depth understanding of one another’s needs, resources and expectations related to research and action. Jacklin and Kinoshameg have argued that in order to be successful in CBPR, researchers “must unlearn the expert role they have been entrenched in” [40] and the I-RREACH tool can facilitate this process. Furthermore, implementation researchers require education not only by listening during the CBPR process but also more formally by learning about topics such as community history and cultural safety in research [41]. Therefore, research teams should also learn about and critically reflect on broader origins of ill health in their partner communities through the implementation process.

### The need for critical reflection on social realities of partner communities

In Canada, there is considerable evidence that governmental assimilation policies, residential schools and forced changes of lifestyle imposed on Aboriginal people have resulted in much of the disease burden and health inequities in Aboriginal communities seen today [42-44]. In the past, these forms of oppression were touted as being in the best interest of Aboriginal people. Aboriginal perspectives were marginalized and negative outcomes were frequently associated with change imposed by outsiders. Given this negative impact of colonialism, Aboriginal communities often refuse standardized solutions offered or imposed by outsiders, and researchers must be cognizant of these historic facts. However, the consequences of a colonial legacy are often difficult to understand for researchers and representatives of governmental institutions, who are typically deeply entrenched in their own worldviews. Canadian Indigenous scholar Willie Ermine aptly observes that

“One of the festering irritants for Indigenous peoples, in their encounter with the West, is the brick wall of a deeply embedded belief and practice of Western universality. Central to the issue of universality is the dissemination of a singular world consciousness, a monoculture with a claim to one model of humanity and one model of society” [45].

The community stakeholders’ insistence in this study on focusing on a range of factors that may affect the outcomes of interventions beyond the community’s direct control was based on their practice-based knowledge as well as an understanding of the hegemony that Ermine describes. The unique influences that underlie health care barriers and disparities in Indigenous communities are complex and include key factors such as socioeconomic status, racism, cultural and communication differences, rural location [46] as well as related lack of resources for services and lack of incentives for providers to practice in rural areas. This perspective is also supported outside of the Indigenous health literature. For example, the notion that an intervention is affected by many factors beyond the control of the community is described by Tee and colleagues. They argue that even a strongly supported intervention within a health system can be seriously affected by “political, financial, educational, cultural, logistic, anthropological, and emotional” barriers [47]. Trickett and co-workers emphasize the importance of influence of culture, the state, policies and ideologies on community interventions [28].

The situation in communities in low-income countries is similar as shown in a recent systematic review indicating that health equity initiatives face potential barriers from a host of challenges in LMICs, including potential resistance from influential actors whose interests or values could be challenged by the intervention and social processes that exclude special interest groups such as rural communities from decision making or intended benefits of the intervention [48]. The review indicates that “politics, process and power must be integrated into the study of health policies and the practice of health system development”. Conversely, research in South Africa shows the positive impact of meaningfully involving local people including Community Health Workers (CHW) in community-based initiatives designed to develop interventions for hypertension [49].

Finally, our evaluation data supports the theoretical framework and provides preliminary support for the validity of the I-RREACH tool as an appropriate implementation tool from the perspective of community stakeholders. The evaluation data also provided important feedback to the research team on the effectiveness of their approach and their own increasing level of cultural competence and safety. We therefore recommend that the evaluation form be used whenever the I-RREACH tool or an adapted version of it is used. Participants should be provided with several minutes to fill out the form (see Table 2) anonymously and to return forms in blank envelopes in order to reduce the social desirability bias. If participants’ literacy is not sufficient to fill out the form, community helpers are needed to help fill out the forms based on the verbal feedback of each participant (as our team did in Tanzania).

### Limitations

The I-RREACH tool has been successfully used to support the implementation of the DREAM-GLOBAL pragmatic RCT and the related health services changes with formal positive feedback from participants as described in this paper. The DREAM-GLOBAL study is still in its implementation phase, and comprehensive data on recruitment and retention of participating communities, providers and patients is not yet available. Additional time and research is required (1) to conduct a detailed process evaluation to inform best practices for the implementation of the tool; (2) to document how the I-RREACH tool and process enabled understanding of implementation issues that can foster effective relationships between researchers and community stakeholders throughout the span of a project ; and (3) to analyse the specific collaborative, CBPR and inter-professional skills required of the research team members to successfully implement I-RREACH.

## Conclusion

The I-RREACH tool was designed to (1) pinpoint key domains required for dialogue between the community and the research team to facilitate implementation of complex health interventions and research projects and (2) to identify existing strengths and areas requiring further development for effective implementation. I-RREACH has been found to be easily adaptable to diverse geographical and cultural settings and can be further adapted to other complex interventions. Further research should include the potential use of the I-RREACH tool in the development of blue prints for scale up of successful interventions, particularly in low-resource environments. Methodological research is also needed on the ongoing requirements for sustainable community engagement in the implementation of interventions, the CBPR skills and other training required of the academic team as well as the role of ongoing mindful reflection as a method of inquiry in implementation research when the I-RREACH approach is applied to implementation research.

## Additional file

Additional file 1:
**I-RREACH TOOL: Intervention and Research Readiness Engagement and Assessment of Community Health Care.** A tool for improving implementation readiness of researchers, organizations and communities in a complex interventions of hypertension management in the First Nations, Inuit and LMIC home care.
