# Supplementary material for: I-RREACH: an engagement and assessment tool for improving implementation readiness of researchers, organizations and communities in complex interventions
Source: Implement Sci. 2015 May 4;10:64. doi: 10.1186/s13012-015-0257-6 (PMC4424962; doi:10.1186/s13012-015-0257-6)
Supplement: Additional file 1: — I-RREACH TOOL: Intervention and Research Readiness Engagement and Assessment of Community Health Care. A tool for improving implementation readiness of researchers, organizations and communities in a complex interventions of hypertension management in the First Nations, Inuit and LMIC home care. [file 13012_2015_257_MOESM1_ESM.docx]

**Additional file 1:** I-RREACH TOOL: Intervention and Research Readiness Engagement & Assessment of Community Health Care. A tool for improving implementation readiness of researchers, organizations and communities in a complex interventions of hypertension management in First Nations, Inuit and LMIC home care

DREAM GLOBAL I-RREACH TOOL

**Note to Researcher:**
Please ensure that the letter of information and letter of consent has been completed according to the ethics protocol. All interviews should be audio recorded to ensure accuracy.

Interview or Focus Group ID:

Name of Interviewer:

Name of community:

Name of Participant)s):

Position (s):

Date:

Time: Start: Finish:

Additional Comments or Notes by Researcher:

COMMUNITY PROFILE

To be partially completed prior to interviews and focus groups based on community engagement visits. Interviewer will fill in gaps after the interview based on the collected data. Interviewer needs to ensure that these topics are covered or find alternate ways to collect this information.

1. **Name of Community:**
2. **Current Chief and contact info:**
3. **Health Care Lead and contact info:**
4. **Community web site:**
5. **Physician Names and contact info:**
6. **Home and Community Care nursing structure and leadership:**
7. **Local MP and MPP:**
8. **Address and location of clinic space, recreation centers for presentations:**
9. **Current planning efforts around high blood pressure, reducing prevalence:**
10. **Health champions and decisions makers (names and role):**
11. **Reliability of cellular coverage:**
12. **Main cellular service provider(s):**
13. **Health registries and databases:**
14. **First point of contact for help for person with high blood pressure:**
15. **Health programs funding sources:**
16. **Proposals or action plans submitted for funding:**
17. **Other chronic disease management programs/assessments recently set up in community:**

Interview Guide

An interview guide to help facilitate dialogue and discussion of strategic topics related to the implementation of health research and interventions between researchers and key stakeholders in the community

**A. LEADERSHIP**

1. Who are the champions and decision makers in terms of health in your community? How are they involved?

Probe:

- 1. Champions could be community members, community health or home care nurse; or community health workers; decision makers could be health directors or executive directors of organizations in the community.

1. How much of a priority is heart health and high blood pressure in particular to the political leadership in your community? Can be used as a probe
2. Would the political leadership support additional efforts to improve blood pressure in the community? Please explain.

Probe: Examples of additional efforts (non-financial) Note to interviewer, please: cover all areas below)

1. Efforts to redefine the role of the home and community care nurse or community health worker?
2. Efforts to use local health centres?
3. Advertising and (for example) use of Treaty days to promote our high blood pressure programs, or conduct a screening day?

**B**. **COMMUNITY PROGRAMS AND ACTIVITIES AND COMMUNITY AWARENSS OF THESE PROGRAMS**

1. What kind of services are available in your community to address high blood pressure?

Probe:

- 1. Are there any community health promotion programs?
  2. Are there programs based on traditional First Nations approaches to health and wellness that may address high blood pressure?
  3. Is there a prevention program for those at risk of hypertension?
  4. Are there any clinics set up to measure blood pressures? Where are they located? Who takes the blood pressures?
  5. Are there any people who take and or monitor their own blood pressure?
  6. What is the role of the traditional healer(s) or traditional faith keepers (or knowledge holders) in the community? Do we need to meet with them?

1. How long have the programs been present in your community? Is there any planning for additional efforts going on in your community?
2. Aside from treatment, what, if anything, is the community doing to attempt to reduce the prevalence of high blood pressure?

Probe:

- 1. Is there health promotion that talks about sodium reduction, healthy eating, or exercise, stress reduction, and alcohol use?

1. What are the strengths of these programs?
2. What are the weaknesses of these programs?
3. Do your health and social services staff work together to plan and deliver programs and services in your community?
4. Are community members aware of any of these programs for high blood pressure? Do they know what to do to prevent high blood pressure?
5. Are there certain members of the community who do not access the services? Please explain?

Probe:

- 1. Do certain members require special efforts to bring them in for blood pressure measures or to get their medications?
  2. Are there certain beliefs in the community about the services?

**C. KNOWLEDGE, HEALTH LITERACY RELATED TO HYPERTENSION & CHRONIC DISEASES**

How important of a concern is hypertension for community members?

1. Does the political leadership have good information about the importance of hypertension and the related complications (such as stroke, heart attack, end-stage renal disease, blindness, amputation, erectile dysfunction, and dementia)?
2. What local health data are available on this issue in your community?

Probe:

1. What kind of registries and/or databases are there?

**D. HEALTH SERVICES LANDSCAPE**

1. To whom would an individual with high blood pressure first turn for help in your community. Why?
2. How are the current health programs funded? Please explain. (Provincial funding, FNIHB, other sources and who?)
3. Are you aware of any proposals or action plans that have been submitted for funding to address high blood pressure in your community? If yes, please explain.
4. Are there any other chronic disease management programs that have been recently set up, including evaluations/assessments that we should know about?

Probe:

1. Are results of these efforts being used to make changes in programs, activities or policies?

**E. COMMUNITY CLIMATE and READINESS to ADDRESS DREAM GLOBAL OBJECTIVES**

*(Interviewer needs to explain briefly the evidence for reducing high blood pressure, and how this relates to the DREAM Global Objectives)*

1. Do you think the community would be willing to engage in changes in the way… :
2. *…Blood Pressure is managed*? (*Dream GLOBAL Objective 3)*
   - Has the community tried a similar program or initiative in the past (especially programs that involved task shifting and/or increasing involvement of the patient/client in their care) )? If yes: Please tell me what happened? Where and who might we expect resistance/support from? Why? If no: Why not?
3. *…sodium intake is addressed at the community level.* (*Dream GLOBAL Objective 2)*

Probe:

- - Do you think the community is ready to mobilize to address sodium intake at the policy level? For example with local businesses, nutritionists, education needs. Looking at the availability of special diets?

1. *Evaluating the Technologies in Hypertension Detection and Control.* (*Dream GLOBAL Objective 4) Interviewer needs to explain this language*

Probe:

- - How common is the use of text messaging in the community? Are there certain members that use it more than others?
  - What is the cell phone coverage like in your community? Is it reliable? Probes: how often does the service go down? How often do you get a signal that is too weak?

**F. INTERVENTION AND RESEARCH READINESS**

1. What has been your community’s experience with health research/interventions?

Probe:

- 1. What kind of research/interventions are currently underway in the community? How about in the past?
  2. Has the experience been positive? Have there been negative reactions? Please explain?
  3. Is there interest in health research or new interventions?
  4. Is here any reason why your community might hesitate to participate in research or a new intervention?
  5. Is there a local ethics committee or research review committee who support Chiefs and Councils in making decisions around research?

1. What kind of capacity building would you expect to see in the research team? What should the researchers learn about the community? ( eg.: learning about local culture, social determinants of health, customs, communications styles, Indigenous values and traditions etc.)
2. For the last question I want to ask you to imagine you are telling me about the project and your experience with it five years from now. Imagine it was successfully implemented in your community. What do you imagine you would tell me about why the project was successful and why?

Probes: (Note to interviewer: Try to probe for positive outcomes : i.e. what worked and why. Allow the interviewee sufficient time to reflect. Only then prompt. Community education? Relationship with the community leadership, health care providers? Updates? Differences in health status?)

Focus Group Guide

A focus group guide to lead the dialogue and discussion between researchers and community members on community-centered issues related to the implementation of health research and interventions.

**B**. **COMMUNITY PROGRAMS AND ACTIVITIES AND COMMUNITY AWARENSS OF THESE PROGRAMS**

1. How important of a concern is hypertension in this community?

2. What kind of services and programs are available in your community to address high blood pressure?

What are the strengths of these programs?

What are the weaknesses of these programs?

How well known are these programs in the community?

What is the role of the traditional healer(s) or traditional faith keepers (or knowledge holders) in the community? Do people rely on them for their health and well being? Do we need to meet with them?

**C. KNOWLEDGE, HEALTH LITERACY RELATED TO HYPERTENSION & CHRONIC DISEASES**

1. How do people access health ( medical) information in your community? What are the current sources of health education?

Probe:

1. Do they go to their family doctor? Do they ask the community health or home care nurse or community health worker or others?
2. Do the media get involved in educating the community?
3. Do traditional healers (or other traditional helpers or Elders) get involved in education programs?
4. Is there a Telehealth helpline, and if so, do people tend to use it?

**D. HEALTH SERVICES LANDSCAPE**

1. Where would a community member first go for help with high blood pressure? (Probe: why?)

**E. COMMUNITY CLIMATE and READINESS to ADDRESS DREAM GLOBAL OBJECTIVES**

*(Interviewer needs to explain briefly the evidence for reducing high blood pressure, and how this relates to the DREAM Global Objectives)*

1. Do you think the community would be willing to engage in changes in the way… :

a*. blood Pressure is managed.* (*Dream GLOBAL Objective 3)*

b*. Sodium intake is addressed at the community level.* (*Dream GLOBAL Objective 2)*

Probe:

- - Addressing sodium intake at the policy level, with local businesses, nutritionists, education needs. Looking at the availability of special diets?

c*. Evaluating the Technologies in Hypertension Detection and Control.* (*Dream GLOBAL Objective 4) Interviewer needs to explain this language*

Probe:

- - How good is the cell phone coverage?
  - How common is the use of text messaging in the community?
  - Are there certain members that text message more than others?

**F. INTERVENTION AND RESEARCH COLLABORATION CAPACITY**

1. What has been your community’s experience with health research and interventions?

Probe:

- 1. What kind of research/ interventions are being done now in the community? How about in the past?
  2. Has the experience been positive? Have there been negative reactions? Please explain?
  3. Is there interest in health research or new interventions?
  4. Is here any reason why your community might hesitate to participate in research or a new interventions?
  5. Is there a local ethics committee or research review committee who support Chiefs and Councils in making decisions around research?

1. What kind of capacity building would you expect to see in the research team? What should they learn about the community? ( eg.: learning about local culture, SDOH, customs, communications styles, Aboriginal values and traditions etc.)
2. For the last question I want to ask you to imagine you are telling me about the project and your experience with it five years from now. Imagine it was successfully implemented in your community. What do you imagine you would tell me about why the project was successful and why?

Probes: (Note to interviewer: Focus on the positive : i.e what worked and why. Allow the interviewee sufficient time to reflect. Only then prompt. Community education? Relationship with the community leadership, health care providers? Updates? Differences in health status?)
